# Optimization of Polymer Processing: A Review (Part II-Molding Technologies)

**DOI:** 10.3390/ma15031138

**Published:** 2022-02-01

**Authors:** António Gaspar-Cunha, José A. Covas, Janusz Sikora

**Affiliations:** 1 Institute of Polymers and Composites, University of Minho, Campus de Azurém, 4804-533 Guimarães, Portugal; jcovas@dep.uminho.pt; 2 Department of Technology and Polymer Processing, Faculty of Mechanical Engineering, Lublin University of Technology, Nadbystrzycka 36, 20-618 Lublin, Poland; janusz.sikora@pollub.pl

**Keywords:** polymer processing, single screw, twin screw, injection molding, blow molding, thermoforming, optimization, artificial intelligence

## Abstract

The application of optimization techniques to improve the performance of polymer processing technologies is of great practical consequence, since it may result in significant savings of materials and energy resources, assist recycling schemes and generate products with better properties. The present review aims at identifying and discussing the most important characteristics of polymer processing optimization problems in terms of the nature of the objective function, optimization algorithm, and process modelling approach that is used to evaluate the solutions and the parameters to optimize. Taking into account the research efforts developed so far, it is shown that several optimization methodologies can be applied to polymer processing with good results, without demanding important computational requirements. Furthermore, within the field of artificial intelligence, several approaches can reach significant success. The first part of this review demonstrated the advantages of the optimization approach in polymer processing, discussed some concepts on multi-objective optimization and reported the application of optimization methodologies to single and twin screw extruders, extrusion dies and calibrators. This second part focuses on injection molding, blow molding and thermoforming technologies.

## 1. Introduction

Societal and economic requirements, as well as environmental sustainability policies, progressively call for manufacturing technologies that consume fewer resources, yield more performing products, and accept bio-derived materials, even if these often have inferior properties. Given the current worldwide concern with the environmental impact of plastics, there is certainly an impetus towards increasing the performance of polymer processing technologies.

Processing of thermoplastic polymers typically comprises a plasticizing step, followed by melt shaping and cooling. These steps have been extensively analyzed both experimentally and phenomenologically in terms of fluid mechanics, heat and mass transfer, rheology, solid mechanics, polymer physics and chemistry. Physical models, and subsequently mathematical descriptions of the processes, have been used to develop computational modelling routines, which are gradually able to determine with good accuracy the behavior of the various processing routines for specific sets of equipment geometry, operating conditions and material properties [1,2,3,4].

In part one of this work [5], it was shown that the available process modelling routines are often used inefficiently to solve real processing problems, such as setting the operating conditions of a process, defining the profile of a plasticizing screw, balancing the runner system of an injection mold, defining the geometry of the pre-form for blowing a bottle with a specific thickness profile, or identifying the temperature distribution of a sheet for thermoforming a part with a given thickness gradient. Indeed, these powerful simulation tools are frequently exploited on a trial and error basis, requiring from the user the capacity to assess the suitability of each process response and input a better one. Therefore, the authors advocated the formulation of real processing problems as optimization problems.

**Figure 1:**
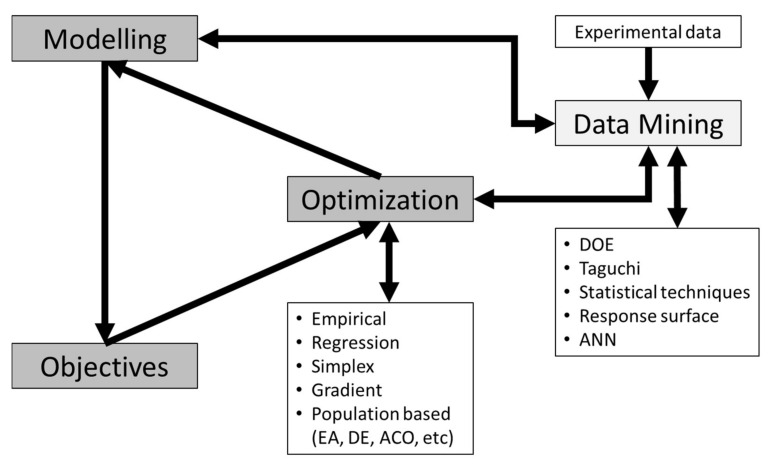
Optimization-based design framework.

As depicted in Figure 1, the optimization approach involves modelling and optimization routines, and routines able to deal with the articulation of the objectives of the optimization. The process modelling routine must be able to handle the decision variables (typically operating conditions and/or geometric parameters) in order to obtain measures of performance (objectives and restrictions). Since most polymer processing problems are multi-objective and multi-constrained [6,7], there is a need to consider the simultaneous existence of several objectives and constraints. Thus, the objective routines must deal with the objectives in such a way that the optimization routines understand, i.e., rank the solutions using a single number. In turn, the optimization algorithms decide about the solutions to be evaluated by the modelling routine, i.e., they define the sets of decision variables to be passed to the modelling routine. This decision is made on the basis of the performance measures of the previously evaluated solution.

Other, often more complex relationships must be also established between the existing and/or generated data and the decision variables, objectives, constraints and environmental parameters (see Figure 1):

(i) Data are generated experimentally and/or computationally. For a given problem, data may be created either while the machine is operating (on-line), or disconnected from the process (off-line) and environmental data may eventually influence the process (e.g., room temperature, humidity). A global and complete portrait of the process can be built by applying, for example, data mining techniques, in order to establish relations between the data, including necessarily objectives and constraints. This type of analysis relies on the use of Artificial Intelligent (AI) models that are able to tackle the decisions about the process;

(ii) The use of environmental data may contribute to obtaining robust solutions, i.e., solutions that perform well for a range of variations of specific design variables. For instance, if the room temperature influences the performance of a specific process, a robustness analysis can be made in order to find a better solution performing satisfactorily under a wider range of room temperatures;

(iii) The multi-objective nature of the problems needs to be considered in a sophisticated way, since the use of simple methods, such as the aggregated sum, often fails even in simple problems [6,7]. Usually, Multi-Objective Optimization Problems (MOOPs) use an optimization algorithm where a specific articulation between the different objectives is made through a single number, designated as a fitness value, which measures the performance of a particular solution. Only optimization algorithms that can tackle various solutions at once can be used as Multi-Objective Optimization Algorithms (MOOA) [6,7];

(iv) Generally, the need of obtaining reliable solutions requires the use of computationally demanding numerical modelling routines. Moreover, during optimization, many modelling runs must be made. An alternative consists in using data mining techniques to develop simpler models, designated by meta or surrogate models. This type of approach relies on the concept of data-driven optimization, or in the use of AI techniques [8,9], and has been previously applied to single screw extruders with innovative designs [10,11,12,13]. 

Table 1 compares the different optimization algorithms that are referred to in this paper. The algorithms were classified based on their adequacy and efficiency, taking into account the important parameters for the optimization problems analyzed here, i.e: dealing with a single objective, finding the global optimum (or a good approximation to it), dealing with a discontinuous objective space and a multi-objective environment, and the flexibility or adaptability to deal with different types of problems.

This work continues the task initiated in part one [5], discussing the application of optimization methods to solve practical problems in polymer processing, specifically involving injection molding, blow molding and thermoforming. The most important contributions to the topic are presented, and the corresponding objective function, process modelling approach, optimization algorithm and decision variables adopted are identified.

## 2. Optimization Algorithms in Polymer Processing

### 2.1. Methodology

The methodology used here is identical to that adopted in Part I, dedicated to extrusion [5]. Thus, the investigation of the open literature on injection molding, blow molding and thermoforming of polymers is based on the following information:

1. Objective function, which can be pursued as a Single Objective (SO), Aggregated Product (AP), Aggregated Sum (AS) or Multi-Objective (MO);
2. Optimization algorithm, which can include Empirical, Simplex, Complex, Regression, Direct, Gradient, Simulated Annealing (SA), Particle Swarm Optimization (PSO), Artificial Bee Colony (ABC), Data Envelopment Analysis (DEA), Ant Colony Optimization (ACO), Evolutionary Algorithms (EA);
3. Process modelling approach, which could be experimental, one-dimensional analytical (1D-A), two-dimensional numerical (2D-N) or three-dimensional numerical (3D-N);
4. Decision variables, i.e., the process parameters to optimize;
5. Other characteristics, related to the process/modelling approach, optimization, etc.

### 2.2. Injection Molding

Injection molding involves two main stages, plasticating the polymer followed by molding and cooling the part inside the mold, which can originate different types of optimization problems. The plasticating step is comparable to plasticating single screw extrusion (see part 1 [5]), the differences being related to the cyclic nature of injection molding, and with the axial screw displacement during plastication and injection. In the case of the molding and cooling phases, the design of the cavity(ies), gate, runner system, and cooling channels, as well as defining the operating conditions can be tackled as optimization problems. Table 2 summarizes the many publications dealing with the optimization of these aspects. As expected, they embrace designing the screw of the plasticiating unit, balancing the runner system, locating the gate, outlining the cooling system, or setting the operating conditions.

Screw design will be discussed first, given its similarity with the design of screws for single screw extrusion, as discussed earlier [5]. This will be followed by an analysis of the literature in terms of the type of objective function, i.e., single objective, aggregation function, multi-objective, and optimization algorithm. 

#### 2.2.1. Plasticating Unit (Screw Design)

Verbraak and Meijer [14] made a major contribution to screw design for injection molding by evaluating experimentally the performance of different screw geometries (conventional three zone screws with different compression ratios, screws containing pineapple, Maddock, or Egan mixing sections, static mixers as add-ons, and/or combinations of these solutions). They took individually into account the maximization of distributive and dispersive mixing, and of the plasticating capacity, and the minimization of melt temperature differences. In order to optimize injection molding screws considering together the operating conditions (screw speed) and screw geometry (channel depth and screw length), Huang [15] applied a grey relational analysis (a data analysis technique) based on the Taguchi method, using a commercial process modelling software, and evaluating the performance in terms of plasticating rate and melt temperature uniformity. Wang et al. [16] applied EA to design screws taking into account the length of the feed and compression zones, the depth of the metering zone and the flight thickness. An ANN was trained based on 3D numerical modelling results defined by the Taguchi method, and the performance was evaluated using the weighted sum of four objectives (maximize output, minimize melt temperature variation at the screw tip, minimize specific mechanical energy and minimize length for melting).

#### 2.2.2. Single Objective Optimization

Concerning single objective optimization of injection molding, Seow and Lam [17] optimized the runner system layout in order to obtain a uniform pressure balance, by changing the thickness of the different flow paths using a trial-and-error procedure. Lee and Kim [18] optimized the injection velocity, cooling and packing times, packing pressure, melt temperature, and mold coolant temperature, with the aim of minimizing part warpage. The complex method was employed, which is a modification of the simplex method to take constraints into account. 

Some authors optimized the process using regression analyses based on experimental results. Chang and Faison [19], Feng et al. [20] and Tang et al. [21] defined the operating conditions with a view to minimizing shrinkage. The last two studies used regression analysis and ANN based on experimental data. Ahmad et al. [22] applied the Taguchi method to analyze experimental data and find the operating conditions that minimize shrinkage. Mukras [23] proposed a framework for optimizing the operating conditions that minimize cycle time while assuming volumetric shrinkage and warpage as constraints. The operating conditions were experimentally related with the objective and constraints by means of the Kriging model (also known as Gaussian process regression, which is an interpolation method based on a Gaussian process), and the selection of the optimal solution was made graphically.

The above regression techniques have been used recently but based on 3D numerical modelling results. Chen et al. [24], aiming to optimize nine operating conditions whilst assuming warpage as an objective and shrinkage as a constraint, applied the response surface method (data analysis) combined with nonlinear programming. Huang et al. [25] linked the Taguchi method with a grey relational analysis to find the best operating conditions (melt and mold temperatures, injection pressure and time, holding pressure) taking individually into account the minimization of warpage and of temperature distribution. The way the optimization was performed seems somewhat unclear since the grey relation analysis is merely a data analysis method.

The gradient method has also been used together with 3D numerical modelling results. Smith et al. [26] optimized both the operating conditions and gate location that minimize the filling time. Lam and Seow [27] applied the same strategy to perform cavity balancing, while Lam and Jin [28] determined the operating conditions that minimized the standard deviation of the flow path or filling time. Pirc et al. [29] designed the mold cooling channels (lengths and diameters) in order to minimize the maximum polymer temperature using sequential quadratic programming (SQP).

Simultaneously, some authors applied more elaborated algorithms, e.g., Simulated Annealing (SA), Evolutionary Algorithms (EA) and Artificial Bee Colony (ABC), to optimize injection molding, although based on a single objective function. Zhai et al. [30] applied EA with 2D modelling and the gradient method with 3D modelling to find the gate location that minimized the injection pressure. These algorithms were also used by Qiao [31] to optimize the location of the cooling channels that minimize the standard deviation of the cavity surface temperature. Li et al. [32] used simulated annealing to optimize the gate location, aiming to reduce warpage.

Single objective optimization using EAs targeted mainly the definition of the operating conditions. Ye and Wang [33] optimized the gate location and the operating conditions with the aim of maximizing the pressure at the end of the filling step. Shi et al. [34] defined the operating conditions that minimize the maximum shear stress in the mold cavities. Lam et al. [35] determined the operating conditions and geometry of the cooling channels that minimize the standard deviation of the cavity surface temperature. Kurtaran et al. [36] optimized the operating conditions in order to minimize warpage, while Ozcelik and Erzurumlu [37,38] and Wu et al. [39] optimized both the operating conditions and several dimensional parameters of the part or mold, also to minimize warpage. With the identical aim, Iniesta et al. [40] used the hybridization of ANN and artificial bee colony (ABC) algorithms. The results were obtained using a 3D numerical modelling software and the ANN was applied to map the objectives as a function of the decision variables. Finally, Changyu et al. [41] defined the operating conditions that minimize volumetric shrinkage. 

#### 2.2.3. Aggregation Function Optimization

Several studies dedicated to optimizing injection molding assumed simultaneously various objectives via an aggregation function and adopted data analysis techniques (for example, the Taguchi method) to define the experimental/computational results to be obtained, and a specific technique to fit the data to a regression model. Singh et al. [42] developed an approach based on the hybridization of Taguchi and desirability function techniques to optimize the operating conditions that minimize cycle time and warpage. The experimental data was fitted to a multiple response surface (data analysis). Sreedharan et al. [43] also applied data analysis to the experimental results based on grey relational and principal component analyses, to generate a response surface and optimize the operating conditions in order to nine objectives that were aggregated in a single function using a weighted sum. Kumar et al. [44] conducted experiments according to the Taguchi L27 Orthogonal Array and analyzed and optimized the response data using the Grey Relational Analysis (an evaluation technique able to solve complex problems for which only incomplete information exists) and a multivariate analysis. The operating conditions that minimize shrinkage, warpage, and surface roughness were identified. Similarly, Yacoub and MacGregor [45] proposed the use of a multivariate statistical analysis to define a response surface taking into account design variables related to material properties and operating conditions to optimize quality measures and the corresponding standard deviations. The optimization was made using a sequential quadratic programming algorithm. This framework was applied to an industrial over-molding process. Kitayama et al. [46] proposed a strategy for taking into consideration various objectives based on the usage of a radial basis function (i.e., a technique able to fit data to a mathematical function), the aim being to set the operating conditions that minimize cycle time and warpage. For that purpose, the authors considered the weighted sum of the objectives and generated successive Pareto fronts after analyzing the previous ones. In reality, this is a data analysis method, since a fit to data is applied. Later, the same optimization methodology was adopted to define the values of the same operating parameters that maximize the minimum weld line temperature and minimize the clamping force [47]. Finally, Moayyedian et al. [48] applied the Taguchi method and a fuzzy analytic hierarchy process to rank the performance of the solutions quantified by a moldability index, which corresponds to a type of weighted sum of the objectives considered. The aim was to determine the operating conditions (melt temperature, gate design, filling and cooling times) that minimize shrinkage, warpage and short shots). 

Gradient optimization techniques were also used in numerous studies with the aim of optimizing operating conditions, gate location and layout of the mold cooling channels. Tang et al. [49] defined the geometry and location of the cooling channels that minimize both the maximum temperature and the temperature gradient, whereas Park and Kwon [50] determined the operating conditions that minimize the average temperature of the molding and the cooling time. Huang and Fadel [51] defined the geometry of the cooling channels that minimize the maximum temperature difference in the part and the cycle time. Shen et al. [52] optimized the gate location with the aim of minimizing the pressure, the filling time difference between different paths, the temperature difference in the part and the percentage of overpacking. Mathey et al. [53] optimized the location and geometry of the cooling channels that maximize the cooling efficiency and rate, using a specific gradient method (sequential quadratic programming). Similarly, Agazzi et al. [54] designed the cooling channels, but in order to minimize temperature and temperature gradient. Shie [55] defined the operating conditions that maximize tensile strength, minimize wear and minimize warpage of the part, considering the product of the objectives. 

Several studies adopted Simulated Annealing, Evolutionary Algorithms or a combination of different techniques. For example, Pandelidis and Zou [56,57] combined gradient and SA methods to define the gate location and the operating conditions that minimized temperature differences in the part, warpage and material degradation. Turng and Peic [58] proposed an integrated tool based on the link between a 3D numerical modelling commercial package and an optimization framework through a single or aggregated objective function, taking into account the relevant constraints, and the possibility of choosing between different optimization algorithms (e.g., gradient, EA, DE and SA). The authors applied this tool to two case studies involving the optimization of the operating conditions, one considering a single objective (minimization of shrinkage in length), the other the weighted sum of two objectives (minimization of cycle time and volumetric shrinkage). Lam et al. [59] unveiled the operating conditions that minimized the maximum shear stress and the maximum cooling time. 

Single objective EAs were used to optimize the part, mold design and operating conditions. Kim et al. [60] aimed at minimizing simultaneously the temperature difference in the part, overpacking and frictional overheating. Chen et al. [61] attempted to minimize both part length and weight, whilst Meiabadi et al. [62] focused on minimizing the maximum pressure, part weight and cycle time. In most cases, the results were obtained using data from a 3D numerical modelling software, except in [61] where experimental results were mapped using an ANN. Finally, Mok et al. [63] optimized the part, mold design and operating conditions, with a view to minimizing maximum wall shear stress, maximum representative shear rate, maximum temperature difference in the part and cycle time.

#### 2.2.4. Multi-Objective Optimization

**Figure 2:**
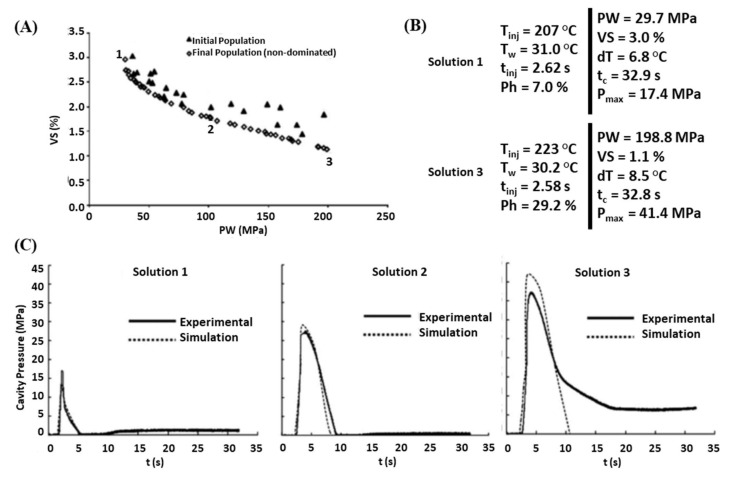
Multi-objective optimization of the operating conditions for injection molding: (**A**) Pareto curves; (**B**) Decision variables and objective values for the optimized solutions; (**C**) Experimental assessment (adapted from [69]).

Alam and Kamal [64,65,66] optimized runners diameters and lengths, as well as the operating conditions that minimized the runner-system volume, cycle time and local part shrinkage differences. Gaspar-Cunha et al. [67] linked an MOEA to a commercial 3D numerical modelling code and determined the operating conditions that induced a specific morphology that was characterized in terms of thermomechanical indices (skin thickness, degree of crystallinity and/or level of molecular orientation). Castro et al. [68] tackled the optimization of the operating conditions and gate location using Data Envelopment Analysis (a multi-objective algorithm) and employing an ANN (which was trained using numerical results) to map the objective as a function of the decision variables. Fernandes et al. [69] applied a MOEA to optimize the operating conditions that minimized temperature differences in the molding at the end of the filling stage, the maximum cavity pressure, the pressure work (i.e., the integral of pressure over time), shrinkage and cycle time. Figure 2 exemplifies the results obtained when minimizing simultaneously volumetric shrinkage (VS) and pressure work (PW). Figure 2A shows the evolution of the Pareto curves along the various generations, displaying a clear improvement. Figure 2B depicts the decision variables and objectives values for two solutions (1 and 3). Not only the trade-off between the two objectives is clear, but it seems that, as expected, holding pressure (Ph) is the most influencing variable. Figure 2C presents a good match between these solutions and the modelling results for the evolution of pressure with time, as well as that solution 2 is more equilibrated concerning the pressure work. Later, using the same MOEA, the locations and diameters of the cooling channels were also optimized, together with the operating conditions, in order to minimize warpage and cycle time [70]. Xu et al. [71] identified the operating conditions that minimized part weight, volume shrinkage and flash, using a multi-objective particle swarm optimization algorithm, and the results given by an ANN trained with experimental data defined by a Taguchi method.

### 2.3. Blow-Molding

Table 3 identifies the reported attempts to optimize blow molding, providing the same type of data as the previous table. As the technology is mostly used for the packaging sector, the focus of optimization is often to reduce the weight of the part in order to reduce the amount of required material while retaining its performance. The discussion is organized according to each specific processing technology, i.e., extrusion blow molding and injection blow molding.

#### 2.3.1. Extrusion Blow Molding

**Figure 3:**
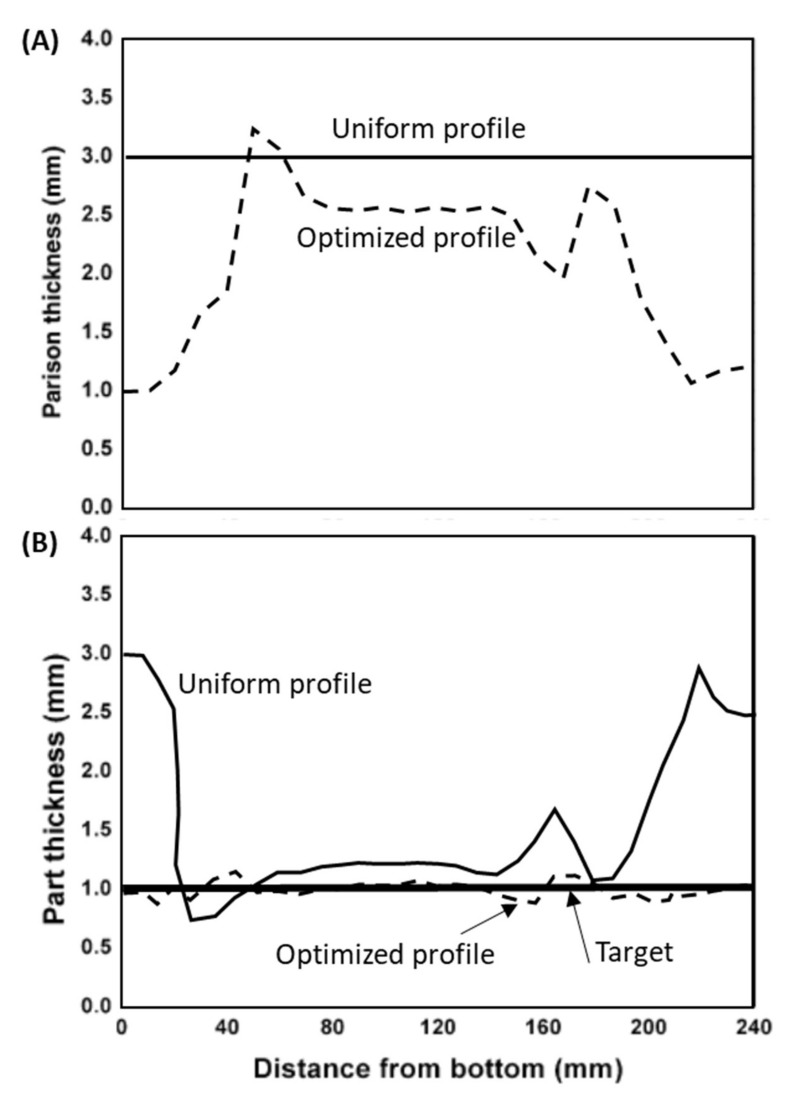
Optimization of the parison thickness profile for extrusion blow-molding: (**A**) Initial uniform and optimized parison profile; (**B**) Part thickness profile after blowing obtained from uniform and optimized parison profiles, together with the target profile (adapted from [82]).

Tahboub and Rawabdeh [72] employed a design of experiments to optimize the operating conditions (screw speed, melt temperature, cooling time, blowing pressure, blowing time and mold temperature) that minimized the variability with time of the volume of the container. Agrawal et al. [73] applied a statistical data analysis based on the Taguchi method and a Grey relational analysis to establish the operating conditions that maximized the compressive strength and volume accuracy of the part. Likewise, Dohare et al. [74] found the operating conditions that maximized haze and clarity, hardness and compressive strength, based on experimental data. DiRaddo and Garcia-Rejon [75] proposed a simple iterative procedure (Newton–Raphson technique) to define the parison thickness profile capable of minimizing the overall thickness variations of the final part, the solutions proposed being obtained with a 3D numerical modelling software. Thibault et al. [76] and Gauvin et al. [77] suggested a multi-disciplinary design optimization (MDO) software environment for optimizing the process. The authors considered two steps: first, the mechanical performance of the blown part was optimized in order to delineate the optimal part thickness distribution; then, the die gap variation minimizing the part thickness variance was established. A single objective was considered—minimizing part weight, together with a constraint regarding a minimum mechanical requirement. Given the significant computation time required by the numerical process modelling, an ANN based on the Taguchi method was trained to define the computational samples and establish relations between the design variables and the objectives; the gradient technique was used in the first step, an EA was applied in the second step [78,79,80]. Later, the same team proposed an EA optimization technique together with an ANN and the Taguchi method, to optimize the die gap variation that minimized part weight while satisfying a few constraints [81]. Huang and Huang [82] determined the parison thickness distribution that produced a part with a predefined thickness profile. For that purpose, an EA together with an ANN were used to evaluate the solutions. Figure 3 illustrates the optimization of the parison thickness profile undertaken. Two parisons with different thickness profiles were used (Figure 3A), one with uniform thickness, the other with an optimized profile (minimizing the differences between the profile obtained and the one targeted (Figure 3B)). The figure compares the thickness profiles of the part obtained from parisons with uniform thickness and with the optimized profile.

#### 2.3.2. Injection Blow Molding

Hopmann et al. [83] determined the thickness distribution of the injected preform that created a blown bottle with a specific wall thickness distribution. The modelling results were obtained using a response surface methodology based on 3D numerical computations, while the optimization was supported by an empirical approach. Bordival et al. [84] applied a Simplex method (the Nelder–Mead optimization algorithm) to define the adequate axial temperature distribution of the preform making a bottle with uniform thickness, using results obtained with a 3D modelling software. The preform thickness profile was also optimized by Biglione et al. [85,86], who attempted to minimize the normalized square root of the difference between the predefined bottle thickness profile and the thickness profile calculated for the solutions by a 3D numerical software. For that purpose, the simplex method and a simple predictor/corrector iterative method were used successively. Demirel [87] used a design of experiments and a response surface method to define, based on experimental data, the operating conditions related to the blowing stage (mold surface temperature and residence time of the bottle in the mold) that optimized the performance of the part (in terms of mechanical and thermal properties). 

Lee and Soh [88] determined the thickness profile of a preform that created a part with a pre-defined thickness contour, while satisfying two constraints (a minimum wall thickness and the absence of undercuts in the inner surface of the preform). The Brent algorithm (a single objective optimization method based on gradient) was applied, and a 3D numerical modelling software supplied the results. Thibault et al. [89] optimized the preform thickness and the operating conditions for injection stretch blow molding, using a gradient-based optimization strategy based on two steps. First, the thickness distribution that minimized the preform weight while satisfying a few constraints (top load, pressurization and vacuum load) was defined, then the operating conditions were optimized. 

**Figure 4:**
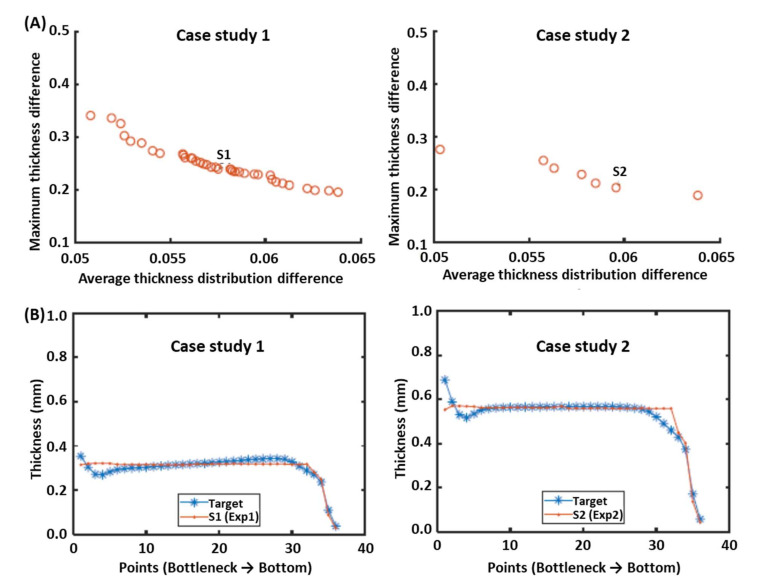
Multi-objective optimization of the preform to obtain the target bottle previously optimized for two case studies: (**A**) Pareto front, average thickness distribution difference between the obtained and the target bottles versus maximum thickness difference between obtained and target bottles; (**B**) comparison between the target bottle profile and the bottle profile obtained for optimized solutions S1 and S2 (adapted from [93]).

Given the multi-objective nature of these problems, MOEA were combined with ANN [90,91,92]. A MOEA was applied to find the best solutions, i.e., to obtain the best trade-off between material usage and mechanical properties, whereas ANN was used to represent thickness distributions. The process was modeled using the ANSYS commercial numerical code. The aim was to determine the best thickness profile for the bottle that assured the desired mechanical properties with minimal material usage. Recently, Pinto et al. [93] found the best thickness profile for the preform that would produce the bottle that was previously optimized. In Figure 4, Solutions S1 and S2 for two different case studies were selected from the corresponding Pareto fronts (Figure 4A) and the thickness profile of these solutions was compared with the target (desired) profile (Figure 4B).

### 2.4. Thermoforming

The term thermoforming covers several technologies that use pressure (either positive or vacuum), eventually assisted by mechanical means, to force a heated sheet against the contours of a mold. Typically, these processes involve a sequence of interdependent stages (heating a sheet, forcing its deformation against a mold and cooling the part), which are dominated by the thermal (heat conduction, emissivity) and mechanical (extensional deformation and deformation rate capacity) properties of the polymer.

The deformation mechanisms developing in thermoforming techniques may create a significant thickness gradient in the final part, which may affect negatively its performance under service. Therefore, optimization challenges often involve finding the best operating conditions (generally, sheet temperature distribution at the end of the heating stage) and/or mold cavity geometry enabling to obtain, as much as possible, a part with a uniform/specific thickness distribution. Table 4 identifies previous efforts to attain these goals. 

The inverse heating problem consists in defining the temperatures of the heaters panel that will produce a uniform sheet temperature, or a specific temperature distribution, after a given heating time. This was solved by Duarte and Covas [94,95]. Obviously, the temperature of the sheet at the beginning of the forming stage will influence the resulting thickness differences in the part, as it determines the local mechanical response of the polymer. Wang and Nied [96] used a gradient optimization method to define the optimal temperature distribution in the sheet. They started by defining the desired thickness profile, and then used 3D numerical modelling to solve iteratively the system for the temperature field needed to obtain the desired result. The iterations started with a uniform temperature distribution. Bordival et al. [97] applied a gradient method (sequential quadratic programming) coupled to a simple analytical model to define the optimized set of operative parameters that allowed to obtain the optimal temperature of the sheet, using a cost function representing the heat flux uniformity. In a subsequent step, these results were used to compute the contribution of the radiation heating resulting from the interaction between the heaters and the thermoplastic sheet. In the end, the three-dimensional transient heat transfer equation was solved using a volume control method. Chy et al. [98,99] used a conjugated gradient optimization method to solve the same problem. This automatic optimization methodology was applied using a complicated scheme with a high number of inputs and outputs for accurate control of sheet temperatures. The authors concluded that both heat transfer by radiation and conduction have an important role, making this a very complex task. Li et al. [100,101] optimized the process employing a response surface coupled to the D-optimal method. First, a 3D numerical modelling software yielded uniform sheet temperature by achieving a steady-state optimum distribution of heater power. Then, the optimization methodology was used to determine the time-dependent optimal heater input by minimizing the temperature difference across the thickness. Erchiqui et al. [102,103] and Bachir-Cherif et al. [104,105] applied two different meta-heuristic algorithms (simulated annealing and evolutionary algorithms) and adopted a 3D volumetric enthalpy-based computational method to determine the optimal sheet temperature distribution. The same authors also applied a gradient-based technique to the same problem [106].

Several studies focused on the optimization of the sheet deformation stage, with the objective of obtaining parts with as much as possible uniform thickness. Yang and Hung [107] proposed an Inverse Artificial Neural Network (IANN) to define the operating conditions (sheet temperature, vacuum pressure, plug speed and vertical displacement inside the mold) that maximized the part thickness uniformity. The inputs of the ANN (which was trained with experimental data) were the thickness distribution at different part locations, while the outputs were the corresponding operating conditions. A similar strategy was adopted to optimize the operating conditions of polypropylene foam thermoforming [108]. Leite et al. [109,110] applied a response surface methodology to solve the same type of problem. An ANN based on experimental data (consisting of an aggregation of different ways of calculating the difference between the thickness of the parts obtained and the desired thickness profile) related the decision variables with the objective. Sasimowski [111] applied the same strategy to identify the operating conditions (heating time, heater temperature, pre-blow time, vacuum time and cooling time) that created a more uniform thickness distribution in the parts. These contributions used ANN to obtain output values from a set of input experimental data. This procedure is analogous to using a regression model, hence its applicability is restricted to the specific cases studied.

**Figure 5:**
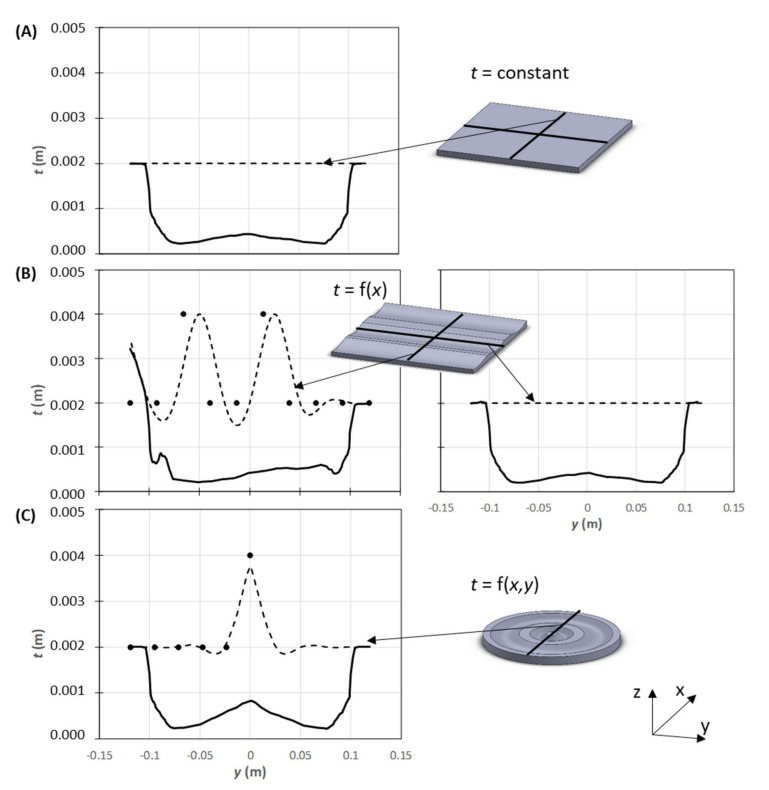
Multi-objective optimization of the initial sheet thickness profile that will induce a uniform thickness distribution in the part and minimize the quantity of material used: (**A**) sheet with uniform thickness; (**B**) sheet with thickness varying transversally to the extrusion direction with a spline shape; and (**C**) sheet with concentric thickness variation with spline shape. The dashed lines represent the sheet profile generated from the points in (**B**,**C**), the continuous line represents the thickness profile of the part (adapted from [112].

The above methods optimized separately the individual process steps. However, since the input of one stage depends on the outcome of the preceding one, only assuming their interdependency enables a truly efficient process optimization. Gaspar-Cunha et al. [112] used a multi-objective evolutionary algorithm to define the initial sheet thickness profile that will induce a uniform thickness distribution in the part, while minimizing the quantity of material used. Three optimization conditions were equated to produce the same part: (i) sheet with uniform thickness; (ii) sheet with thickness varying transversally to the extrusion direction with a spline shape; and (iii) sheet with concentric thickness variation with spline shape. The solutions were evaluated using a 3D numerical modelling software. Figure 5 presents three optimized solutions for each case. 

## 3. Conclusions

This review discussed the application of optimization methodologies to the most important thermoplastics processing technologies. The existence of a strong interdependence between objective function, optimization algorithm and data collecting (i.e., experimental or computational data) is evident.

When approaching real processing situations as optimization problems, at least two objectives must be considered in order to reach valuable conclusions, as different aspects are to be analyzed simultaneously, i.e., processing problems are multi-objective.

Selecting an optimization algorithm depends on the problem features, and whether the goal is to optimize one or several objectives. Even if a few objectives exist, any type of optimization algorithm can be applied by simply aggregating them into a single objective. However, aspects such as data scarcity, the possibility of generating data during the optimization, and the time required to obtain such data, should also be taken into consideration. 

The results produced using statistical, ANN, response surface and other regression methods are of limited value, as only a few parameters are used, either in the domain of the decision variables or in that of the objectives. Statistical approaches require a high amount of data, thus a significant number of simulations is required in order to define a good response surface, but without guaranteeing that the optimization will not be stuck in a local optimum. Moreover, the optimization is intrinsically connected to the specific situation under consideration. If either the polymer properties, the operating conditions and/or the equipment geometry change, a completely new analysis must be carried out. Thus, regression methods are not very distinct from trial-and-error procedures where the optimization progresses with the help of the user. 

Artificial Intelligence (AI) techniques, such as EAS, ANN, DE and data mining seem efficient approaches to address the optimization of complex polymer processing problems, as they are able to provide continuous or discrete solutions, use available data through a learning process, and deal efficiently with multiple objectives.

An optimization run is only successfully concluded when the preferences of a decision maker are introduced in a given process step, and the best solution has been chosen. The decision maker can be a human or a machine (which can link optimization with Artificial Intelligence). In this way, AI technologies used in other fields can be applied in engineering problems. For example, *innovization* consists in establishing effective rules between all the variables of the system (decision variables and objectives), from the data obtained after the run of a MOEA. This is a further step towards developing computational tools that are able to provide informed solutions to the engineer based on data analysis - they are designated as data-driven optimization methodologies. In a certain way, the latter is a form of returning to situations where only a limited quantity of data is available, since the new AI technologies allow, within some limits, to perform optimizations based on a limited amount of data, experimental or computational. This is certainly a trend that still requires further research. For example, Trinh et al. [113] applied these types of techniques to chemical product engineering, suggesting some guidelines for further research.

## Figures and Tables

**Table 1:** Classification of the optimization algorithms adequacy and efficiency: “---”, not adequate; “--”, very inefficient; “-”, inefficient; “+”, adequate; “++”, efficient; “+++”, very efficient.

Algorithm	Single Objective	Global Optimum	Discontinuous Objective Space	Multi-Objective	Flexibility
Empirical	+	---	---	---	---
Simplex	+	--	---	---	---
Complex	++	--	--	---	---
Regression	++	--	---	---	--
Direct	+	---	---	---	---
Gradient	+++	-	-	---	---
Simulated Annealing	+++	+	+	++	+
Particle Swarm Optimization	+++	+	+	+++	+
Artificial Bee Colony	+++	+	+	+++	+
Data Envelopment Analysis	++	+	+	++	-
Ant Colony Optimization	+++	+++	+++	+++	++
Evolutionary Algorithms	+++	+++	+++	+++	+++

**Table 2:** Previous publications on the optimization of injection molding (SD—screw design, GL—gate location, OC—operating conditions, MD—mold design, CC—cooling channels, RS—runner system, CB—cavity balancing, PG—part geometry).

Objective Function	Optimization Algorithm	Modelling Approach	Decision Variables	Other Characteristics	Authors (Year), Reference
SO	Empirical	Experimental	SD	Variousgeometries	Verbraak and Meijer (1989) [14]
SO	Regression	3D-N	SD		Huang (2016) [15]
WS	EA	3D-N	SD		Wang et al. (2020) [16]
SO	Empirical	3D-N	CB		Seow and Lam (1997) [17]
SO	Complex	3D-N	OC		Lee and Kim (1995) [18]
SO	Regression	Experimental	OC	DOE	Chang and Faison (2001) [19]
SO	Regression	Experimental	OC	ANN	Feng et al. (2006) [20]
SO	Regression	Experimental	OC	ANN	Tang et al. (2007) [21]
SO	Regression	Experimental	OC	Taguchi	Ahmad et al. (2019) [22]
SO	Regression	Experimental	OC	Kriging model	Mukras (2020) [23]
SO	Regression	3D-N	OC		Chen et al. (2010) [24]
SO	Regression	3D-N	OC		Huang et al. (2015) [25]
SO	Gradient	3D-N	GL + OC		Smith et al. (1998) [26]
SO	Gradient	3D-N	CB		Lam and Seow (2000) [27]
SO	Gradient	3D-N	GL		Lam and Jin (2001) [28]
SO	Gradient	2D-N	CC	SQP	Pirc et al. (2008) [29]
SO	EA + Gradient	2D-N + 3D-N	GL		Zhai et al. (2005) [30]
SO	EA + Gradient	2D-N	CC		Qiao (2006) [31]
SO	SA	3D-N	GL		Li et al. (2007) [32]
SO	EA	3D-N	OC + GL		Ye and Wang (1999) [33]
SO	EA	3D-N	OC	ANN	Shi et al. (2003) [34]
SO	EA	3D-N	OC + CC		Lam et al. (2004) [35]
SO	EA	3D-N	OC		Kurtaran et al. (2005) [36]
SO	EA	3D-N	CC		Ozcelik and Erzurumlu (2005) [37]
SO	EA	3D-N	OC	ANN	Ozcelik and Erzurumlu (2006) [38]
SO	EA	3D-N	OC + RS + PG		Wu et al. (2011) [39]
SO	ABC	3D-N	OC	ANN	Iniesta et al. (2013) [40]
SO	EA	3D-N	OC	ANN	Changyu et al. (2007) [41]
AS(2)	Regression	Experimental	OC	Taguchi	Singh et al. (2018) [42]
AS(8)	Regression	Experimental	OC		Sreedharan et al. (2019) [43]
AS(3)	Regression	Experimental	OC	Gray Rel. Anal.	Kumar et al. (2019) [44]
AS (20)	Regression	Experimental	OC	SQP	Yacoub and MacGregor (2004) [45]
AS(2)	Regression	3D-N	OC	RBF	Kitayama et al. (2017, 2018) [46,47]
AS(3)	Regression	3D-N	OC	Fuzzy analysis	Moayyedian and Mamedov (2019) [48]
AS(2)	Gradient	3D-N	CC		Tang et al. (1997) [49]
AS(2)	Gradient	3D-N	OC		Park and Kwon (1998) [50]
AS(2)	Gradient	3D-N	CC		Huang and Fadel (2001) [51]
AS(4)	Gradient	3D-N	GL		Shen et al. (2004) [52]
AS(2)	Gradient	3D-N	CC	SQP	Mathey et al. (2004) [53]
AS(2)	Gradient	3D-N	CC		Agazzi et al. (2010) [54]
AP(3)	Gradient	3D-N	OC		Shie (2008) [55]
AS(3)	Gradient + SA	3D-N	GL and OC		Pandelidis and Zou (1990, 1990) [56,57]
SO + AS(2)	Gradient + EA + DE + SA	3D-N	OC		Turng and Peić (2002) [58]
AS(2)	Gradient+EA	3D-N	OC		Lam et al. (2006) [59]
AS(3)	EA	3D-N	OC		Kim et al. (1996) [60]
AS(2)	EA	Exp. + ANN	OC		Chen et al. (2007) [61]
AS(3)	EA	3D-N	OC	ANN	Meiabadi et al. (2013) [62]
AS(4)	EA	3D-N	OC + MD + PD	ANN	Mok et al. (2001) [63]
MO(3)	EA	3D-N	OC + RS		Alam and Kamal (5 April 2003 [64,65,66]
MO(3)	EA	3D-N	OC	morphology	Gaspar-Cunha et al. (2005) [67]
MO(5)	DEA	3D-N	OC + GL	ANN	Castro et al. (2007) [68]
MO(4)	EA	3D-N	OC		Fernandes et al. (2010) [69]
MO(2)	EA	3D-N	OC+CC		Fernandes et al. (2012) [70]
MO(3)	PSA	Experimental	OC	Taguchi + ANN	Xu et al. (2012) [71]

**Table 3:** Previous publications on the optimization of blow molding. Decision variables: OC—operating conditions; PaTP—parison thickness profile; PTP—part thickness profile; PfTP—preform thickness profile; PfTemP—preform temperature profile; DGO—die gap opening.

Objective Function	Optimization Algorithm	Modelling Approach	Decision Variables	Processing Technology	Reference
SO	Regression	Experimental	OC	Extrusion	Tahboub and Rawabdeh (2004) [72]
SO	Regression	Experimental	OC	Extrusion	Agrawal et al. (2012) [73]
SO	Regression	Experimental	OC	Extrusion	Dohare et al. (2018) [74]
SO	Gradient	3D	PaTP	Extrusion	Diraddo and Garcia-Rejon (1993) [75]
SO	Gradient	3D	PTP	Extrusion	Thibault et al. (2001) [76]
So	Gradient	3D	PTP + DGO	Extrusion	Gauvin et al. (2003) [77]
SO	Gradient+EA	3D	PTP + DGO	Extrusion	Yu et al. (2002, 2004) [78,79]
SO	Gradient+EA	3D	PTP + DGO	Extrusion	Hsu et al. (2004) [80]
SO	Gradient	3D	DGO	Extrusion	Yu and Juang (2010) [81]
SO	EA	3D	PTP	Extrusion	Huang and Huang (2007) [82]
SO	Empirical	3D	PfTP	Injection	Hopmann et al. (2015) [83]
SO	Simplex	3D	PfTP	Injection	Bordival et al. (2009) [84]
SO	Simplex	3D	PfTP	Injection	Biglione (2015) [85]
SO	Simplex	3D	PfTP	Injection	Biglione et al. (2016) [86]
SO	Regression	Experimental	OC	Injection	Demirel (2017) [87]
SO	Gradient	3D	PfTP	Injection	Lee and Soh (1996) [88]
SO	Gradient	3D	PfTP + OC	Injection	Thibault et al. (2007) [89]
MO(3)	EA	3D	PTP	Injection	Denysiuk et al. (2017, 2019) [90,91]
MO(3)	EA	3D	PTP	Injection	Pinto et al. (2019) [92]
MO(3)	EA	3D	PfTP	Injection	Pinto et al. (2021) [93]

**Table 4:** Previous publications on the optimization of thermoforming (decision variables: OC—operating conditions; TempD—temperature distribution; SThD—sheet thickness distribution).

Objective Function	Optimization Algorithm	Modelling Approach	Decision Variables	Other Characteristics	Reference
SO	Empirical	1D-N	TempD		Duarte and Covas (1997, 2002) [94,95]
SO	Gradient	3D-N	TempD		Wang and Nied (1998) [96]
SO	Gradient	1D-A	TempD		Bordival et al. (2005) [97]
SO	Gradient	3D-N	TempD		Chy and Boulet (2010) [98]
SO	Gradient	3D-N	TempD		Chy et al. (2011) [99]
SO	Regression	3D-N	TempD		Li et al. (2008) [100]
SO	Regression	3D-N	TempD		Li et al. (2010) [101]
SO	SA + EA	3D-N	TempD		Erchiqui et al. (2011 [102])
SO	SA + EA	3D-N	TempD		Bachir-Cherif et al. (2015) [103]
SO	SA + EA	3D-N	TempD		Erchiqui (2018) [104]
SO	Gradient	3D-N	TempD		Bachir-Cherif et al. (2018) [105]
SO	SA + EA	3D-N	TempD		Bachir-Cherif (2019) [106]
SO	IANN	Experimental	OC	Plug assisted	Yang and Hung (2004) [107]
SO	IANN	Experimental	OC	Plug assisted	Chang et al. (2005) [108]
SO	Regression	Experimental	OC	Vacuum	Leite et al. (2018, 2018) [109,110]
SO	Regression	Experimental	OC	Vacuum, pre-blow	Sasimowski (2018) [111]
MO(2)	EA	3D-N	SThD	Plug assisted	Gaspar-Cunha et al. (2021) [112]

## Data Availability

Not applicable.
